# Classification of Drugs Listed in Iranian Pharmacopoeia Based on Impact on Driving Performance 

**Published:** 2019-08

**Authors:** Mostafa Farahbakhsh, Faramarz Pourasghar, Homayoun Sadeghi Bazargani

**Affiliations:** ^*a*^Research Center of Psychiatry and Behavioral Sciences, Tabriz University of Medical Sciences, Tabriz, Iran.; ^*b*^Tabriz Health Services Management Research Center, Tabriz University of Medical Sciences, Tabriz, Iran.; ^*c*^Road Traffic Research Center, Tabriz University of Medical Sciences, Tabriz, Iran.

## Abstract

According to studies, drugs based on their Pharmaco-dynamics and pharmacokinetics can affect driving performance. There are clear conclusions about some medications and in other drugs have not yet reached definitive conclusions. There are two international experiences regarding the classification of drugs based on their impact on driving performance. These projects are DRUID (Driving Under the Influence of Drugs and medicines) and ICADTS projects. This study conducted to classify Iranian pharmacopeia according to drugs effects on driving performance. The projects divided the drugs used in the treatment of diseases into three categories in terms of traffic safety. The project first identified and explored similar experiences in the world by reviewing scientific resources. For the medicines that their effects were determined in the existing classifications, their impact category were extracted from DRUID and ICADTS systems. For drugs that have different categories in two systems or unidentified their effects on these classifications, Drug side effects, especially in areas of attention, cognition, sleep, vital signs were extracted from pharmacology and other related textbooks. With using appropriate keywords, the studies and evidences about these drugs effects on driving were assessed. At an expert meeting, with review of side effects and scientific evidences, the impact of these drugs on driving performance was determined. Finally, the effect of drugs on driving performance was classified into three groups with no or minimal, moderate and severe effects. Based on these classification results, a mobile application was designed and prepared for ordinary people. In this application, for any drug their effect category, side effects and prevention strategies were explained.

**Keywords:**

Drugs, Driving, Traffic Safety, Driving Behavior

